# The Crystal Structure of the Orphan Nuclear Receptor NR2E3/PNR Ligand Binding Domain Reveals a Dimeric Auto-Repressed Conformation

**DOI:** 10.1371/journal.pone.0074359

**Published:** 2013-09-12

**Authors:** M. H. Eileen Tan, X. Edward Zhou, Fen-Fen Soon, Xiaodan Li, Jun Li, Eu-Leong Yong, Karsten Melcher, H. Eric Xu

**Affiliations:** 1 Laboratory of Structural Sciences, Van Andel Research Institute, Grand Rapids, Michigan, United States of America; 2 Department of Obstetrics & Gynecology, National University Hospital, Yong Loo Lin School of Medicine, National University of Singapore, Singapore, Singapore; 3 Key Laboratory of Regenerative Biology, Guangzhou Institute of Biomedicine and Health, Chinese Academy of Sciences, Guangzhou, China; 4 Van Andel Research Institute/Shanghai Institute of Materia Medica Center, Chinese Academy of Sciences-Key Laboratory of Receptor Research, Shanghai Institute of Materia Medica, Chinese Academy of Sciences, Shanghai, China; University of Oldenburg, Germany

## Abstract

Photoreceptor-specific nuclear receptor (PNR, NR2E3) is a key transcriptional regulator of human photoreceptor differentiation and maintenance. Mutations in the NR2E3-encoding gene cause various retinal degenerations, including Enhanced S-cone syndrome, retinitis pigmentosa, and Goldman-Favre disease. Although physiological ligands have not been identified, it is believed that binding of small molecule agonists, receptor desumoylation, and receptor heterodimerization may switch NR2E3 from a transcriptional repressor to an activator. While these features make NR2E3 a potential therapeutic target for the treatment of retinal diseases, there has been a clear lack of structural information for the receptor. Here, we report the crystal structure of the apo NR2E3 ligand binding domain (LBD) at 2.8 Å resolution. Apo NR2E3 functions as transcriptional repressor in cells and the structure of its LBD is in a dimeric auto-repressed conformation. In this conformation, the putative ligand binding pocket is filled with bulky hydrophobic residues and the activation-function-2 (AF2) helix occupies the canonical cofactor binding site. Mutations designed to disrupt either the AF2/cofactor-binding site interface or the dimer interface compromised the transcriptional repressor activity of this receptor. Together, these results reveal several conserved structural features shared by related orphan nuclear receptors, suggest that most disease-causing mutations affect the receptor’s structural integrity, and allowed us to model a putative active conformation that can accommodate small ligands in its pocket.

## Introduction

Nuclear receptors (NRs) constitute a large family of DNA-binding transcription factors that modulate gene expression involved in an extremely broad spectrum of physiology. The complete human genome contains 48 nuclear receptors, which include classic endocrine receptors, adopted orphan receptors, and orphan receptors [1]. Over the years, much focus has been placed on the classic nuclear receptors, such as androgen, estrogen, and glucocorticoid receptors, whose physiological regulation by small molecule ligands has made them among the most successful drug targets. In contrast, the class of orphan receptors, for which no ligand was known when cloned, remains of enormous interest, as their physiological roles are still emerging.

NR2E3 is an orphan nuclear receptor that is highly expressed in photoreceptor cells [2-4] and plays pivotal roles in photoreceptor development, differentiation, and survival [4-17]. The human retina contains ~5% cone and ~95% rod photoreceptor cells. Rods contain a single type of visual pigment, rhodopsin, for high-sensitivity low light vision. In contrast, human cones contain one of three alternative pigments (S-, M-, and L-opsins), each, which respond to short (S), medium (M), and long (L) wavelengths for color and bright-light high-resolution vision. In concert with other transcription factors, NR2E3 has a dual role to consolidate the rod fate of rod precursor cells: NR2E3 is associated with the promoters of both cone-specific genes, including the cone opsin genes, to repress their transcription, and rod-specific genes, including rhodopsin, to activate their transcription. The default state of photoreceptor precursor differentiation appears to be formation of S-cone cells, which is inhibited in rod precursor cells by NR2E3 repressor function [8,9,10,11,12,15]. NR2E3 is not only expressed during photoreceptor cell differentiation, but rather is continuously expressed at high levels in mature retina [13,14], consistent with its proposed additional neuroprotective function.

NR2E3-null mutations cause retinal degeneration and lack of color vision in mice [6,7,11,14], whereas various point mutations have been associated with enhanced S-cone syndrome (ESCS) [8,18-22]. ESCS is characterized by increased numbers of S-cones and reduced or undetectable rod function [8,18], which causes slow, progressive retinal degeneration that can ultimately lead to complete blindness [19]. Apart from ESCS, NR2E3 mutations have also been associated with clumped pigmentary retinal degeneration, recessive and autosomal dominant retinitis pigmentosa, and Goldman-Favre disease [7,19,23-33]. NR2E3 is therefore a potential target for the treatment of different eye diseases. NR2E3 has also been linked to cancer as it regulates Phosphatase and TENsin homolog (PTEN) [34], p53 [35], and estrogen receptor α [36].

One key question for NR2E3 is whether its activity is regulated by ligands. While attempts to search for physiological ligands have been futile for this orphan nuclear receptor, 13-*cis* retinoic acid and synthetic compounds (11A and 11B) were identified as agonist ligands of NR2E3 [37]. These compounds both stimulate Gal4 DNA Binding Domain–NR2E3 LBD-dependent reporter gene expression and reduce the interaction of the LBD with the co-repressors Nuclear receptor Co-Repressor (NCoR) and Silencing Mediator for Retinoid or Thyroid-hormone receptors (SMRT) in yeast two hybrid experiments [38]. Despite these efforts in ligand identification, NR2E3 functions predominantly as a ligand-independent transcriptional repressor in most cell based assays, for which the underlying structural mechanism remains largely unclear. In this study, we solved the crystal structure of an apo-NR2E3 LBD, which reveals an auto-repressed dimeric conformation that is required for NR2E3 repressor functions.

## Results

### Crystal structure of the NR2E3 LBD in an autorepressed conformation

**Figure 1:**
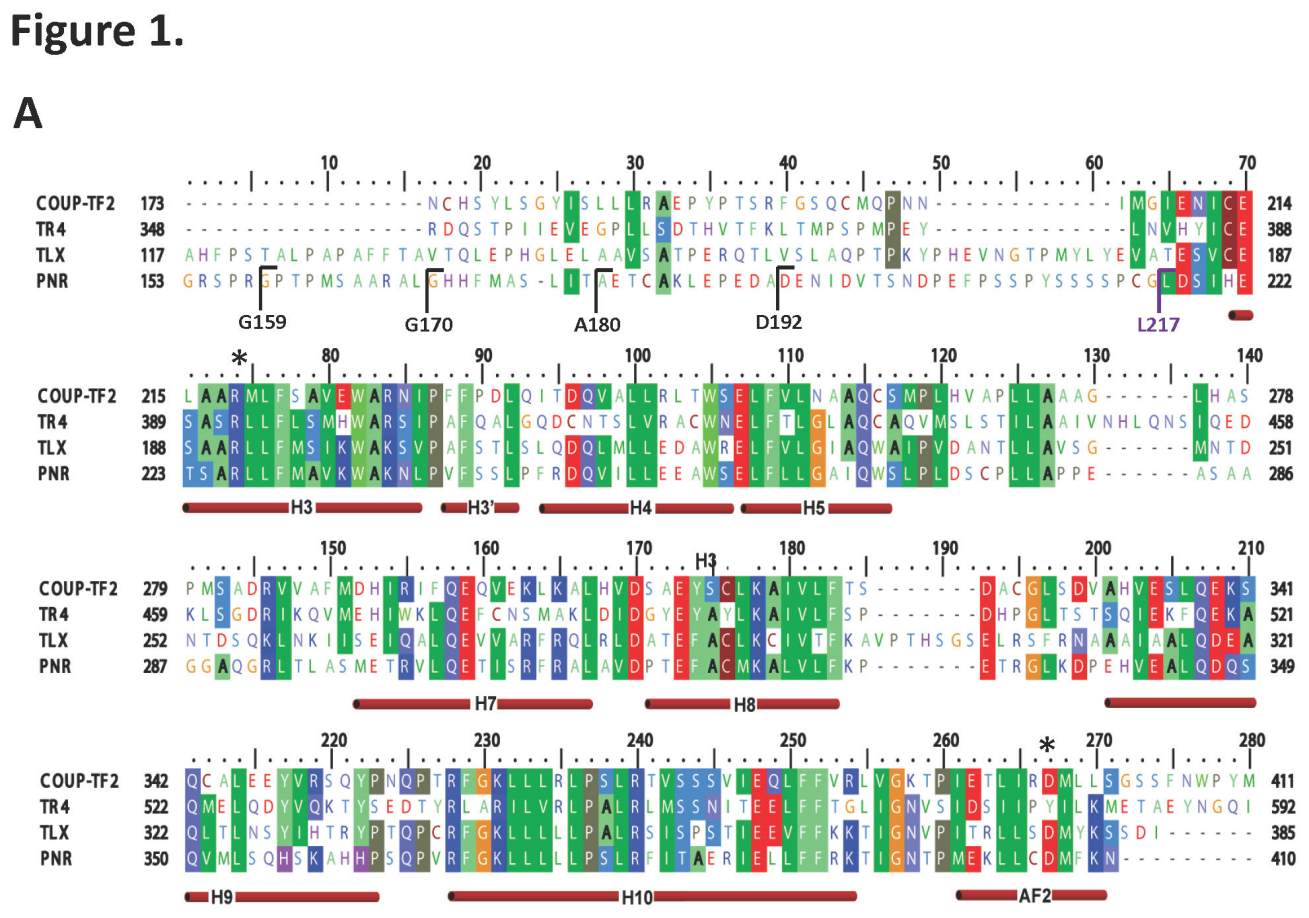
Sequence alignment of NR2E3 with other orphan receptors (COUP-TFII, TR4 and TLX) in the NR2 subgroup family and predictions on their secondary structure. Constructs were designed with various N-terminal start points (G159, G170, A180, D192 and L217). Predicted α-helices are indicated as red cylinders below the sequences. Asterisks indicate the two charge clamp residues that are important for the correct positioning of coactivator LXXLL motifs in the cofactor binding groove.

NR LBDs are characterized by a canonical fold, typically comprising 12 α-helices, of which the N-terminal helices are much less conserved and defined. To determine the domain boundaries of the NR2E3 LBD, we designed four LBD expression constructs with variations at the N-terminus, starting at either G159, G170, A180, or D192 of full length NR2E3 (Figure 1). When expressed as His_6_ Glutathione S-Transferase (GST)- or His _6_Sumo-fusion proteins, all of them were poorly soluble and unstable. Expression with a non-cleavable N-terminal Maltose Binding Protein (MBP) tag and C-terminal His_6_ tag yielded soluble proteins that gave crystals, but these crystals did not diffract X-rays. Based on the poor behavior of these proteins and the weak conservation of the NR2E3 LBD N-terminus, we predicted that the NR2E3 LBD might not have an N-terminal helix 1, which is a characteristic of the related orphan nuclear receptor Testicular Receptor 4 (TR4). The N-terminal region of TR4 lacks a helical structure and instead adopts a long flexible loop that extends to the canonical helix 3 (Figure 1) [39].

**Figure 2:**
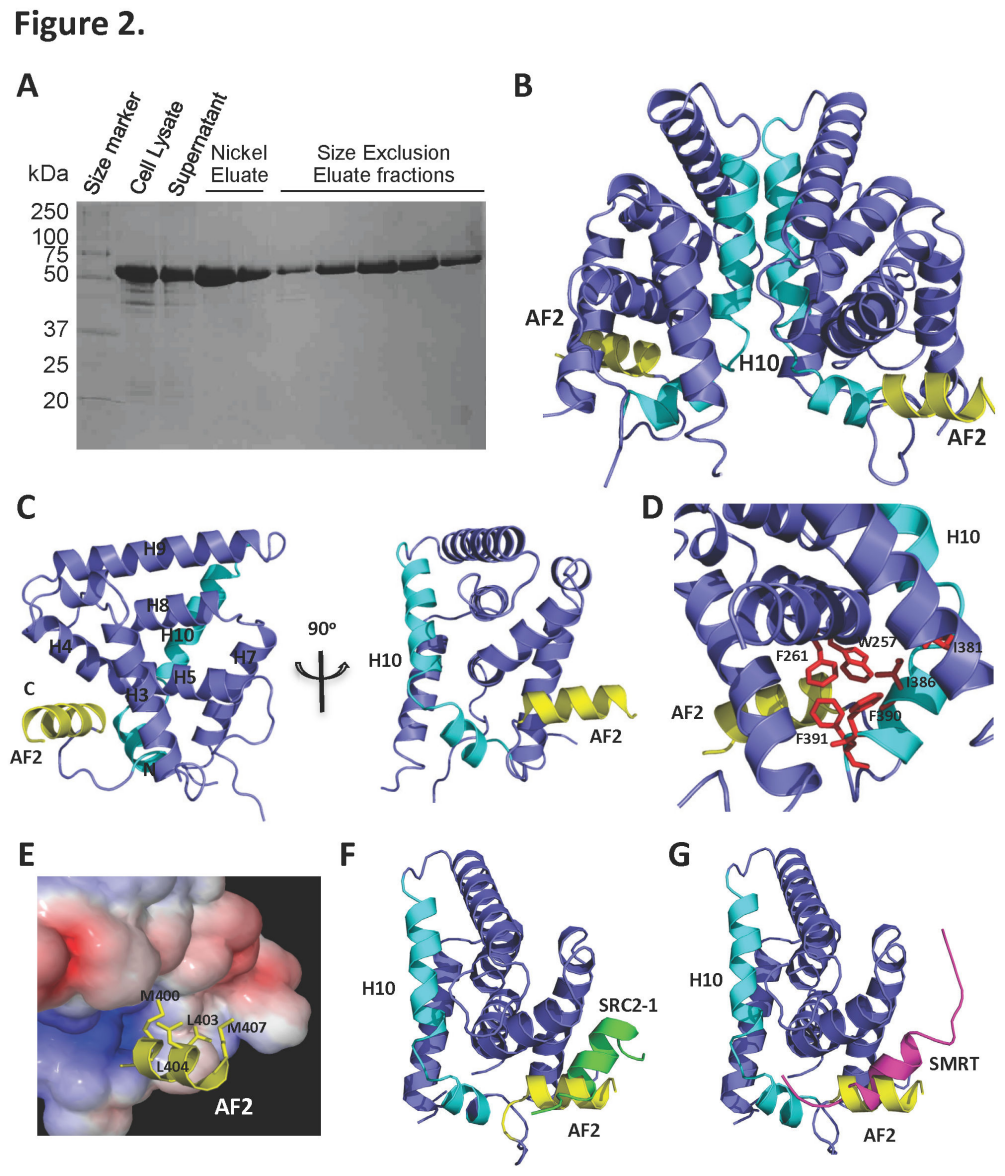
Crystal structure of the NR2E3 LBD is in an autorepressed conformation. **A**. Purification of the MBP**-**NR2E3 LBD-His_6_ fusion protein. The molecular weight of full length MBP–NR2E3LBD-His_6_ is 63.6 kDa. **B**. Overview of the NR2E3 dimer. Each monomer is colored purple, with helix 10 (H10) colored cyan and activation function domain 2 (AF2) colored yellow. **C**. Front and side views of the NR2E3 LBD monomer. The secondary structure assignment is labeled according to nuclear receptor testicular receptor 4 (TR4). **D**. The ligand binding pocket space within the bottom half of the NR2E3 LBD is occupied by large hydrophobic side chains (shown in red stick presentation). **E**. Hydrophobic interactions of the NR2E3 AF2 helix within the cofactor binding site. Positively charged surfaces are shown in blue, negatively charged surfaces in red, and the uncharged, hydrophobic groove in white. **F**. Overlay of the NR2E3 LBD structure with the SRC1 LXXLL motif (in green) from the RXR structure (1K74). **G**. Overlay of the NR2E3 LBD structure with the SMRT LXXIIXXXL corepressor motif (in magenta) from the antagonist-bound PPARα structure (1KKQ).

We thus designed an expression construct of NR2E3(217-410), which lacks the N-terminal region that corresponds to the flexible loop in TR4, as a fusion protein with a non-cleavable N-terminal MBP tag and a C-terminal non-cleavable His_6_ tag. This fusion protein was expressed and purified to homogeneity in the absence of ligand (Figure 2A). The MBP tag when fused to a target protein can stabilize the target protein and greatly facilitate crystal formation of the fusion proteins [40-43]. As it is in our case, we readily crystallized the MBP-NR2E3 fusion protein in the P2 _1_2 _1_2 space group and solved its structure at a resolution of 2.8 Å by using the MBP and TR4 structures as the molecular replacement models (Figure 1B). The statistics of data collection and structure refinement are shown in Table 1.

**Table 1:** Statistics for crystallographic data collection and structure refinement.

PDB code: 4LOG	
**Data collection**	
Resolution (Å)	30-2.70
Space group	P21212
Unit cell, a, b, c (Å)	89.43, 184.94, 85.97
Reflection, unique/total	38683/264280
Completeness (%)	96.4 (76.8)
Redundancy	6.8 (4.5)
R_merge_ (%)	8.5 (65.5)
Intensity, I/σ	20.2 (2.0)
**Structure determination and refinement**	
Resolution Range (Å)	30-2.7
Protein residues	686
No. of water molecules	180
R/R_free_ (%)	27.0/31.1
r.m.s.d. bonds (Å)	0.0097
angles (°)	1.161
Mean B value (Å^2^)	60.5

The crystal structure reveals a dimeric arrangement of the NR2E3 LBD (Figure 1B). Each monomer consists of a canonical antiparallel three-layer α-helical sandwich fold comprising 8 alpha helices (H3-5, H7-H10, and AF2), with a disordered region between helix 5 and helix 7 (Figures 1, 2B, and 2C). The dimer interface is a parallel coiled-coil formed by the helices 10 of both monomers (Figure 2B). Similar to the structures of the ligand-free orphan NRs TR4, Rev-erb, NUclear Receptor Related-1 protein (NURR1), Chicken Ovalbumin Upstream Promoter-Transcription Factor II (COUP-TFII), Dosage-sensitive sex reversal, and Adrenal hypoplasia critical region, on chromosome X, gene 1 (DAX-1) [39,44-47], the apo NR2E3 LBD lacks a ligand-binding pocket. Instead, the C-terminal part of helix 10 bends and collapses into the space that corresponds to the ligand binding pocket in other NR. This space is filled with hydrophobic and aromatic residues (W257, F261, I381, I386, F390 and F391) whose bulky side chains protrude into the cavity (Figure 2D) and, in this conformation, do not provide enough space to allow ligand binding.

The kink in helix 10 and the subsequent collapse of the binding pocket of NR2E3 allows the AF2 helix, which follows helix 10, to bind in the cofactor binding site of the LBD. Hydrophobic interactions stabilize the AF2 binding in this position, with residues M400, L403, L404 and M407 in the AF2 helix forming Van-der-Waals interactions with residues from helix 3 and 4 (Figure 2E). As a result of the occupation of the cofactor-binding site, the AF2 helix would block the binding of either a coactivator or a co-repressor to the LBD and would therefore be expected to inhibit the receptor’s activity (Figure 2F and 2G). Thus, the collapsed ligand-binding pocket and the blocked coactivator binding groove indicate that the apo NR2E3 LBD adopts an autorepressed conformation.

### AF2 is important for NR2E3 repression

**Figure 3:**
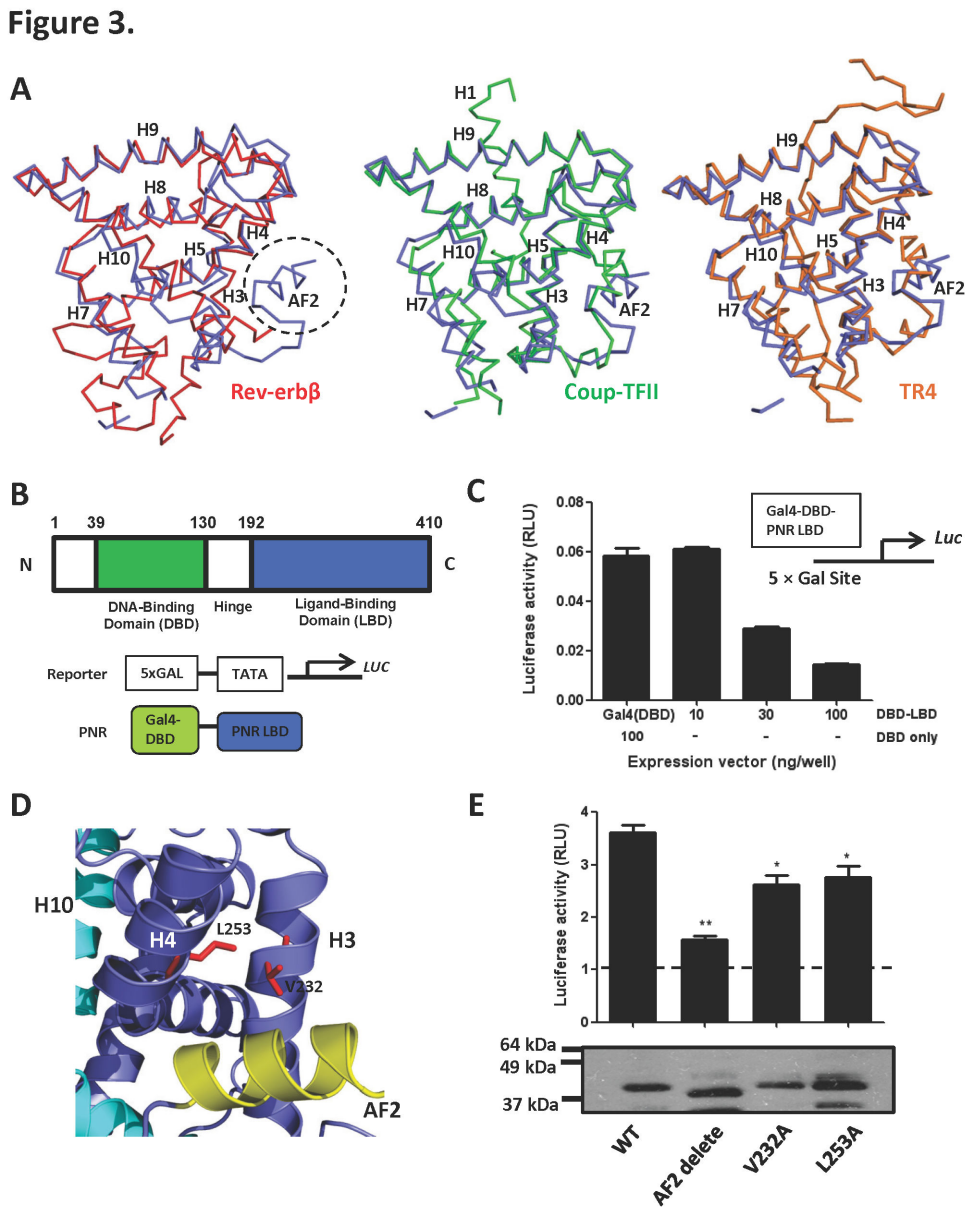
Activation function domain 2 is important for NR2E3 repression. **A**. Superposition of the alpha-C helix traces of the NR2E3 monomer (blue) with those of Rev-erbβ (red), Coup-TFII (green), and TR4 (orange). **B**. Schematic presentation of the domain structure of NR2E3 and of the transfection constructs. **C**. Gal4DBD–NR2E3LBD is a transcriptional repressor. AD293 cells were transiently transfected with either Gal4DBD (100 ng) or Gal4DBD–NR2E3LBD (10, 30, 100 ng) expression plasmids. Transcriptional activity was measured as luciferase activity. Luciferase activity was derived by normalizing firefly luciferase values to *Renilla* luciferase values, which was used as internal transfection control. **D**. The corepressor hydrophobic binding groove is formed by helix 3 (H3), helix 4 (H4), and activation function 2 (AF2). **E**. Effects of AF2 deletion and V232A and L253A mutations on NR2E3 repressor activity (top). Below: Relative Gal4DBD–NR2E3LBD expression levels determined by anti-Gal4DBD immunoblot. The molecular weight of full length Gal4DBD–NR2E3LBD is 42 kDa.

Similar autorepressed features have been observed in the structures of the ligand-free orphan nuclear receptors Rev-erbβ (2V0V), COUP-TFII (3CJW), and TR4 (3P0U) [39,45,47] (Figure 3A). Superposition of the NR2E3 LBD to that of the above orphan nuclear receptors shows a clear similarity with root mean square deviations (rmsd) of 1.88 Å (131 C^α^ atoms), 1.42 Å (147 C^α^ atoms) and 1.38 Å (153 C^α^ atoms) respectively. There are noticeable differences between Rev-erbβ and NR2E3 in the position of helix 7 and in the very C-terminus, as Rev-erbβ lacks the activation function 2 helix (Figure 3A). In the absence of an AF2 motif, helix 11 provides a structural platform for the binding of the co-repressor NCoR1, which is crucial for the constitutive repressive activity of Rev-erbβ [47]. COUP-TFII and TR4 LBDs are overall very similar to that of NR2E3, but differ in their AF2 position (Figure 3A). We have previously shown that the AF2 helix is important for the transcriptional activation function of both COUP-TFII and TR4 [39,45]. Since binding of canonical NR corepressors and coactivators occurs in the hydrophobic groove formed by helices 3, 4, and AF2 [48], we were interested to test whether mutations and deletion of this region can affect NR2E3 transcriptional repression.

In order to correlate the observed structural features with NR2E3 function, we expressed the NR2E3 LBD (residues 192-410, Figure 3B) as a chimeric protein fused to the DNA binding domain (DBD) of the yeast transcription activator Gal4. This construct was transiently transfected into AD293 cells and its effect was tested on a Gal4-responsive reporter plasmid using a luciferase assay (Figure 3B). In agreement with previous studies, this construct functioned as a transcriptional repressor [2] [37] [49] and reduced basal luciferase reporter activity in a dose dependent manner (4-5 fold reduction with 100 ng of NR2E3 expression vector; Figure 3C).

To test the role of the AF2 helix for NR2E3 activity, we expressed a C-terminally truncated LBD (residues 192-400) that lacks the entire AF2 helix. Surprisingly, the AF2-truncated LBD is expressed at the same level as the full length LBD, yet has strongly reduced repression activity (Figure 3E), suggesting that the AF2 helix is required for NR2E3 transcriptional repressor activity. To pinpoint the amino acids involved in this interaction, we created a series of site-directed mutations to substitute hydrophobic residues (I220, L228, L229, V232, L235) along helix 3 and 4 against alanine and tested these constructs in cell-based activation assays. Our data suggest that amino acids V232 on helix 3, L253 on helix 4, and the AF2 helix are important for transcriptional repression by this receptor. Since the AF2 helix blocks access to the canonical cofactor-binding site, these results further suggest the intriguing possibility that the AF2 helix in the apo conformation sterically clashes with coactivator binding, while NR2E3-specific corepressors may still be able to associate with NR2E3 via additional cofactor-binding surfaces on the receptor (see Discussion).

### NR2E3 transcriptional repression activity requires the formation of a functional dimer

**Figure 4:**
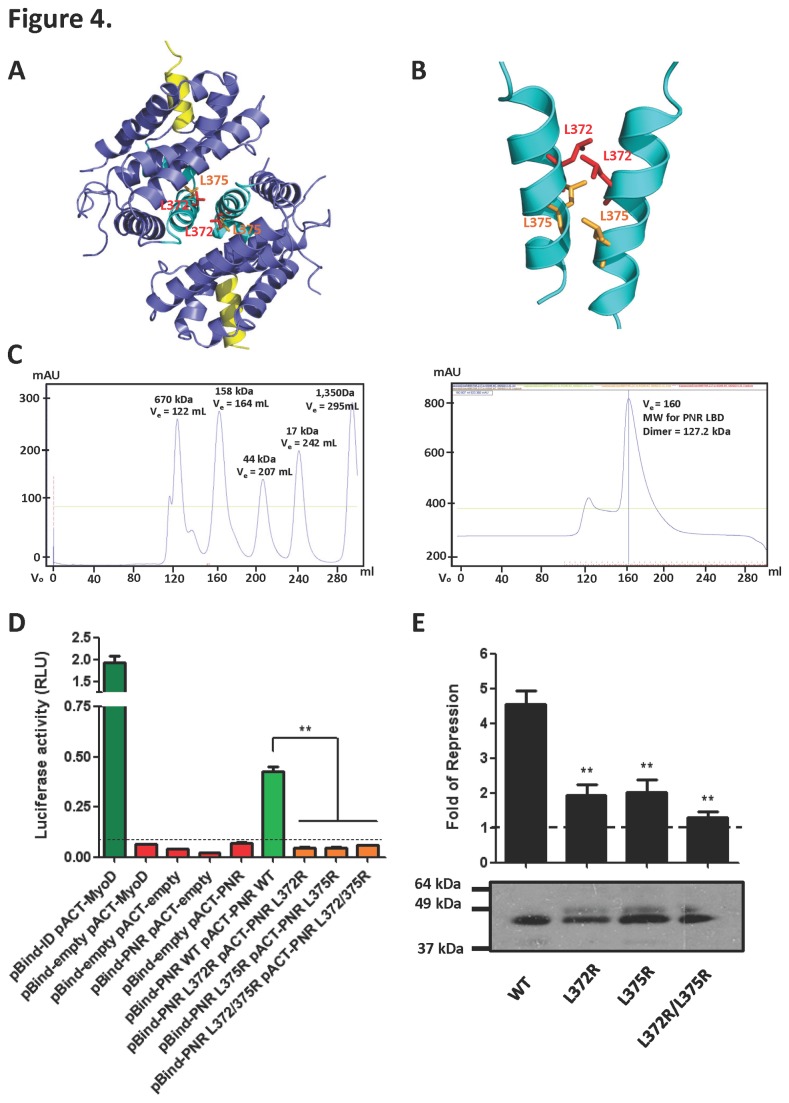
NR2E3 transcriptional repression activity requires the formation of a functional dimer. **A**. Top view of the NR2E3 LBD dimer, showing the close interaction of L372 and L375 (red and orange stick models, resp.) from the helices 10 (cyan) in the dimer interface. **B**. A close-up view of the helices 10 in the dimer interface. **C**. Size Exclusion Chromatography for Bio-rad Protein Standard (left) and MBP-NR2E3 LBD (right). **D**. Mutation of helix 10 coiled coil interface residues abolished LBD dimerization in a mammalian two hybrid assay. Reporter gene activation by Gal4DBD–NR2E3LBD and VP16AD–NR2E3LBD wildtype and mutant expression plasmids is shown as bar graph. Cells cotransfected with pBIND-Id and pACT-MyoD were used as positive controls. **E**. Effects of the mutations L372R, L375R, and the double mutation L372R/L375R on NR2E3 repression activity (top). Below: Expression levels of wildtype and mutant Gal4DBD–NR2E3LBD determined by immunoblotting.

The formation of dimeric LBDs is one of the hallmarks observed in many NRs. Importantly, the NR2E3 LBD also forms dimers in solution as shown by size exclusion chromatography (Figure 4C). In order to determine the role of the NR2E3 dimer configuration in NR2E3 repressor function, we made single or combined mutations to replace two key residues of the coiled coil dimer interface with arginine (L372 and L375; Figure 4A and 4B). These residues are conserved in the related NRs COUP-TFII, TR4, and TLX (Figure 1) and predicted to compromise or abolish LBD dimer formation. To test this prediction, we cloned wildtype and mutant PNR LBD (amino acids 192-410) into pBind-Gal4 and pVP16 vectors for mammalian two hybrid assays. As shown in Figure 4D, the mutant LBDs failed to produce specific reporter gene activity, suggesting that both the L372 and the L375 residue are important for dimer formation. We then tested whether NR2E3 repressor activity is affected when NR2E3 dimer formation is disrupted. As shown in Figure 4E, single mutations of L372 and L375 to arginine reduced NR2E3 repression ability by >50%, and the double mutant L372R/L375R retained only 20-30% of the activity of the wild-type receptor. These results suggest that transcriptional repression by NR2E3 requires the formation of a functional LBD dimer, consistent with other studies that showed the functional form of full length NR2E3 is a dimer [2] [49], and indicate that L372 and L375 are crucial residues for forming the hydrophobic dimerization interactions.

### Computer-modeling predicts a ligand pocket in the active conformation of the NR2E3 LBD

**Figure 5:**
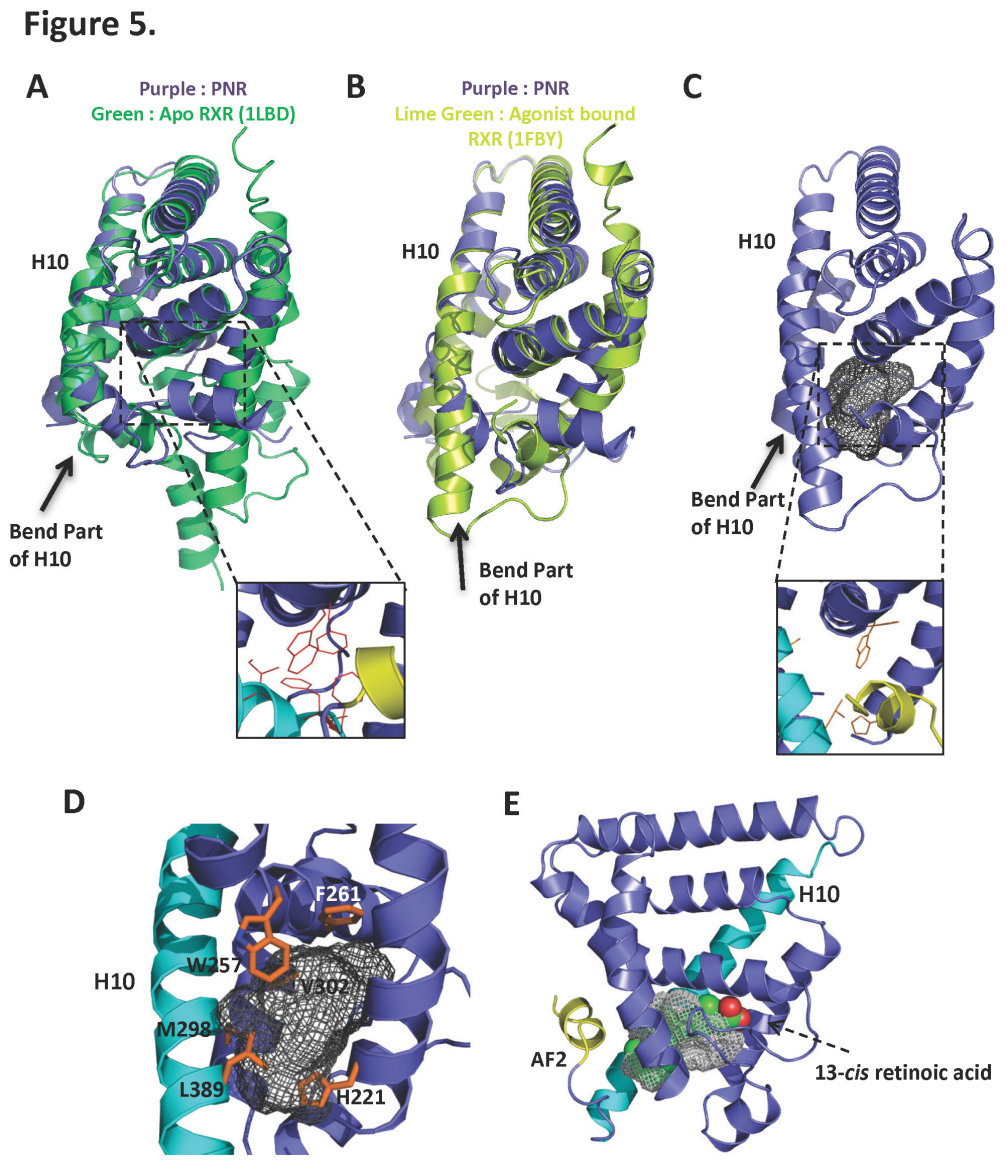
Computer model of the active conformation of the NR2E3 LBD reveals a putative open ligand-binding pocket. **A**. Overlay of the apo LBDs of NR2E3 (purple) and RXR (green). **B**. Overlay of the LBDs of apo NR2E3 (purple) and agonist-bound RXR (lime green). The main difference between the ligand binding pockets lies in the extension of helix 10 in agonist-bound RXR. **C**. Computer model (SWISS Model) of the NR2E3 LBD in an active conformation based on the agonist-bound RXRα structure, with helix 10 extended. A ligand binding pocket of 578 Å^3^ was found in this conformation. The pocket volume and the surface (grey mash) were calculated using the program VOIDOO. **D**. Close-up of the potential ligand binding pocket in the active model of the NR2E3 LBD and its surrounding residues. **E**. 9-*cis* retinoic acid (ball model), which has limited NR2E3 agonistic properties [37], fits well into the modeled ligand binding pocket.

One of the goals of determining the NR2E3 structure was to determine whether it has a ligand-binding pocket and if its activity is regulated by a hypothetical ligand. As mentioned above, the ligand binding pocket of the structure we have solved is packed with hydrophobic residues and space in the pocket is further limited by the kink at the C-terminal end of helix 10. The structure of the related nuclear receptor Retinoid X Receptor (RXR/NR2B1) LBD has been solved both in the apo state [50] as well bound to the RXR agonist 9-*cis* retinoic acid [51]. To illustrate possible conformational changes toward a hypothetical active NR2E3 LBD structure, we overlaid the NR2E3 LBD structure with both the apo- (Figure 5A) [1LBD] and agonist-bound (Figure 5B) [1FBY] RXR LBD structures. The core of the apo-NR2E3 structure is largely similar to that of the apo-RXRα structure, but differs substantially from the structure of the RXR LBD bound to its agonist 9-*cis* retinoic acid, in which helix 10 is fully extended (Figure 5B). Notably, 13-*cis* retinoic acid, an isomer of 9-*cis* retinoic acid, exhibits at high concentrations limited NR2E3 agonist properties [37].

To probe whether the NR2E3 LBD can accommodate a hypothetical ligand, we modeled an active conformation of the NR2E3 LBD based on the structure of the agonist bound-RXRα LBD (PDB code: 1FBY), which has 39% sequence identity with the NR2E3 LBD [2]. The model of activated NR2E3 contains a ligand-binding pocket that is surrounded by the hydrophobic residues H221, F261, W257, M298, V302 and L389. The pocket has a calculated size of 578 Å^3^ (Figure 5C and 5D), a space adequate to accommodate small compounds. While 13-*cis* retinoic acid can be nicely fitted in the active conformation pocket (Figure 5D), the space is not able to accommodate compound 11A, a synthetic NR2E3 activator [37]. This result is consistent with a recent proposal that compound 11A does not bind directly to the NR2E3 pocket [52].

### Disease-causing mutations in the NR2E3 LBD predominantly affect receptor structural integrity

**Figure 6:**
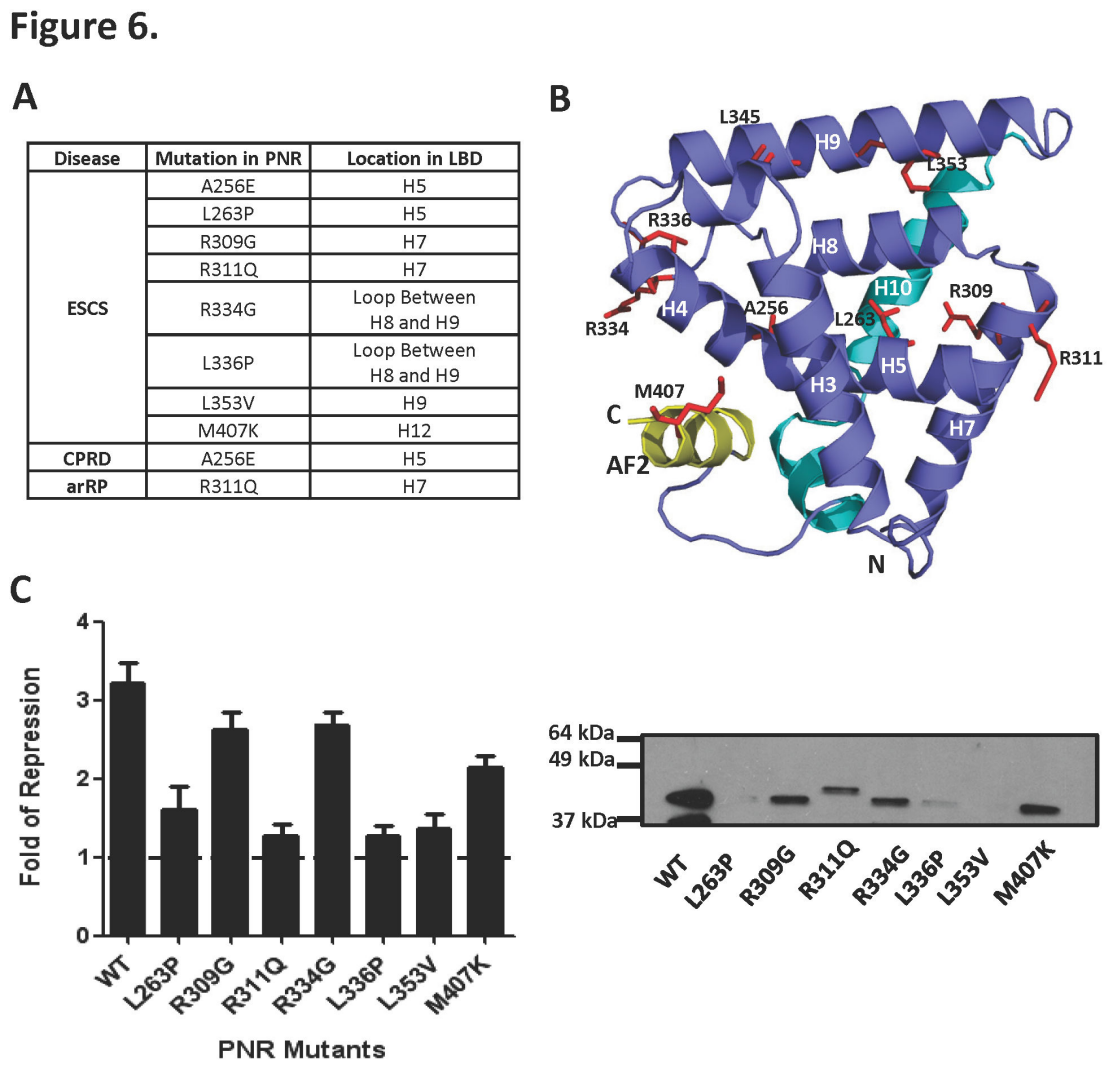
Structural analysis of disease-causing mutations in NR2E3. **A**. List of reported NR2E3 mutations found in patients with various eye diseases. ESCS: enhanced S-cone sensitivity syndrome, CPRD: clumped pigmentary retinal degeneration, ARRP: autosomal recessive retinitis pigmentosa. **B**. NR2E3 LBD mutations found in patients mapped on the receptor. The view is presented similar to the orientation shown in Figure 2C. **C**. Effects of eye disease mutations on NR2E3 repressor activity. Left panel: Reporter gene expression of Gal4DBD–NR2E3LBD wildtype and mutant proteins. Right panel: Expression levels of wildtype and mutant Gal4DBD–NR2E3LBD determined by immunoblotting.

Single amino acid mutations in NR2E3 have been associated with various eye diseases, but the basis by which they affect NR2E3 function is unknown. To gain insight into the function of amino acid residues altered in patients, we mapped residues deposited in the Leiden Open Variation Eye Disease Database (www.LOVD.nl/eye) on the LBD crystal structure (Figure 6A and 6B) and determined the effect of these mutations on NR2E3 repressor activity in a cell-based assay (Figure 6C). As described previously [53] [54], and confirmed here in another cell line, mutants L263P, R311Q, R336P, L353V, and M407K strongly reduced the transcriptional repressor activity of the NR2E3 LBD, consistent with these mutants being disease-causing. Patient mutations mapped mostly to helices 5, 7 and 9 (Figure 6A and 6B). Surprisingly, altered amino acids were not located in regions involved in receptor dimerization, ligand binding, or the canonical corepressor binding site at the AF2 site, regions that we would expect to affect NR2E3 transcriptional repressor activity. In order to test if the lack of repressor activity was a result of poor protein stability, we analyzed protein expression levels by immunoblotting. Expression of the NR2E3 mutants L263P, R336P, and L353V was almost abolished and expression of the remaining mutants R309G, R311Q, R334G and M407K was strongly reduced, suggesting that these mutations mainly affect NR2E3 function by reducing protein stability.

## Discussion

NR2E3 has been one of the last few nuclear receptors for which no structural information exist and that are difficult for crystallization studies. In order to crystallize the NR2E3 LBD in the absence of ligands, we had to remove the N-terminal region of the LBD and fuse the truncated LBD to an MBP tag via a short and stiff linker. Fusion of MBP with a short linker has previously been used by us and others to promote crystallization of various target proteins [40,41,55-59]. Recently, it has been shown that T4 lysozyme and apo-cytochrome b _562_RIL fused N-terminally [60] or inserted into a cytoplasmic loop [61-63] of several 7-transmembrane receptors promoted crystallization by replacing flexible regions and by providing crystallization contacts, suggesting fusion with a stable tag may be a general strategy for crystallizing difficult proteins.

Use of these engineering approaches allowed us to determine the crystal structure of the NR2E3 LBD. The structure of the receptor in the ligand- and cofactor-free state revealed an autorepressed dimeric conformation. We confirmed dimerization by size-exclusion chromatography and mammalian two-hybrid assays and showed that dimerization occurs through formation of a coiled coil that involves key hydrophobic interactions by L372 and L375 of helix 10. Mutations of these residues abolished LBD dimerization in the two-hybrid assay and impaired repressor function of the LBD, providing support for the importance of LBD dimerization for NR2E3 activity.

The auto-repressed conformation of the NR2E3 LBD is reminiscent of the conformations of the ligand-free LBDs of other orphan NRs, such as COUP-TFII, Rev-erbβ, and TR4. In each of these LBDs, the corepressor binding site is blocked by the AF-2 helix and the hydrophobic part of helix 10 projects into the ligand binding pocket to stabilize the inactive LBD conformation. While these orphan receptors share similar folds, they are regulated by diverse mechanisms. This diversity is consistent with the lack of conservation of LBD surface residues, which are key drivers for protein-protein interactions, resulting in interactions with different sets of transcription factors and coregulatory complexes that lead to different functions and activities.

Nuclear receptors carry out their diverse transcriptional functions through the recruitment of coactivators or corepressor [27] that bind to conserved overlapping binding grooves. Since AF2 blocks access to the cofactor binding site in the apo structure of NR2E3 and other orphan NRs, corepressors must either bind to alternative or additional sites of the LBD or they must displace the AF2 helix in order for NR2E3 to function as an active transcriptional repressor. In the latter case, we would expect that AF2 deletion would increase repressor activity, just the opposite of what we have observed. While we cannot exclude the possibility that deletion of the AF2 helix compromised NR2E3 repressor activity simply due to gross LBD misfolding, we think this is unlikely as the expression of the AF2-truncated LBD was unaltered (Figure 3E), in stark contrast to the strongly reduced expression of all disease-associated mutant LBDs (Figure 6C). We therefore favor a model, in which corepressors for NR2E3 can bind to sites other, or in addition to, the canonical cofactor binding site. To date, the best characterized NR2E3 corepressor is Ret-Cor, which functions as specific and directly NR2E3-binding bridging protein to recruit a large corepressor complex that contains histone deacetylases, Sin3A, E2F/Myb associated protein, and NCor [64].

While the function of NR2E3 as a transcriptional repressor is well established, studies revealed that NR2E3 can also work as a transcriptional activator [15]. How NR2E3 can switch from a transcriptional repressor to an activator is currently poorly understood, but may involve three different mechanisms: (i) synergistic interactions with Neural Retina Leucine zipper (NRL), Cone-Rod homeo box (CRX), and Rev-erb α transcription factors [4,11,15,16], (ii) regulation by Protein Inhibitor of Activated STAT3 (PIAS3)-catalysed sumoylation [65], and (iii) binding of currently unidentified physiological agonists. Most NRs are converted from transcriptional repressors to activators by binding of small molecule agonists in their ligand-binding pockets. Potential non-physiological endogenous (13-cis retinoic acid) and synthetic (compounds 11A and 11B) agonists have been identified for NR2E3, but it is not clear if they can directly bind NR2E3 [38,52]. Since the apo NR2E3 LBD in our structure, similar to other ligand-free orphan NRs [39,44,45], lacked an open ligand-binding pocket, we used a modeling approach to probe whether an active NR2E3 LBD conformation could contain a ligand-binding pocket suitable for the binding of small molecule agonists. Our modeled structure suggests that the NR2E3 LBD may be capable to adopt a structure similar to that of RXR bound to its agonist 9-*cis*-retinoic acid, with a ligand pocket of a size and shape suitable to accommodate the weak NR2E3 agonist 13-*cis* retinoic acid. Our structural analysis therefore indicates that NR2E3 may be able to be regulated by small compounds.

Finally, we have mapped disease-associated mutations on the LBD crystal structure. There is a clear discrepancy between our structure obtained by X-ray crystallography and previous NR2E3 LBD structure predictions [23] [53]. The crystal structure therefore provides the first accurate information on location and interactions of amino acid residues altered in disease. Eye disease-associated mutations that we analyzed were distributed over helices 5, 7 and 9 (Figure 6B). These helices are important to maintain the structural integrity of the LBD, and mutations mapped onto these helices likely destabilize the protein as the corresponding mutant proteins display severe to moderate expression defects (Figure 6C).

In conclusion, we have solved the structure of the NR2E3 LBD in an auto-inhibited and ligand-free state, which allowed us to analyze molecular details of NR2E3 repression, including the importance of dimer formation and the unexpected requirement of activation function 2 in the cofactor-binding site for repressor activity. Currently, there is no treatment to restore vision in diseases linked to NR2E3 malfunction. The computer model of an active state NR2E3 LBD suggests that NR2E3 may undergo conformational changes to adopt an unobstructed ligand-binding pocket suitable for the binding of small molecule agonists. The structure presented here may therefore help to understand NR2E3 regulation at the molecular level and may aid in the search for physiological or therapeutic modulators for the treatment of NR2E3-associated eye diseases.

## Materials and Methods

### Protein preparation

The human NR2E3 LBD (NR2E3 residues 217-410) with C275S mutation was expressed as a MBP–NAAAEF linker–NR2E3(217-410)-His_6_ fusion protein from the expression vector pETDuet1 (Novagen). Six liters of BL21 (DE3) cells were grown to an OD_600_ of 1, followed by an induction with 100 µM of isopropyl-beta-D-thio-galactopyranoside (IPTG) at 16 °C. After overnight incubation, cells were harvested and resuspended in 100 ml of extract buffer (200 mM NaCl, 25 mM Tris, pH 8, 10% glycerol, 2 mM β-mercaptoethanol, and 200 µl of saturated PMSF). Cells were lysed using a French Press with pressure set at 1,000 Pa. Lysates were centrifuged at 13,000 rpm for 50 min and the supernatant was loaded onto a pre-equilibrated 5 ml Ni-chelating Hi-Trap column (Amersham Biosciences). The column was washed with 100 ml buffer A (200 mM NaCl, 25 mM Tris pH 8, 25 mM imidazole and 10% glycerol) and the fusion protein eluted with buffer B (200 mM NaCl, 25 mM Tris pH 8, 500 mM imidazole, and 10% glycerol). The eluted proteins was further purified by Superdex 200 gel filtration chromatography (Amersham Biosciences) in 200 mM ammonium acetate, 25 mM Tris, pH 8, 1 mM EDTA, and 1 mM dithiotreitol and concentrated to 5 mg/ml for crystallization.

### Crystallization and data collection

Crystals of the MBP-NR2E3 LBD-His_6_ fusion protein were grown at 20 °C in hanging drops containing 1 µl of the above protein solution and 2 µl of well buffer containing 7% polyethylene glycol 6000 (Sigma), 8% isopropanol, 10 mM Tris(2-carboxyethyl) phosphine (TCEP) and 0.1 M sodium citrate, pH 5.6. Crystals appeared within 3 days and grew to a final size of 100-300 µm on day 7. Crystals were mounted and soaked in the mother liquor solution supplemented with 20% 2,3 butanediol for cryo-protection. All crystals were flash frozen in liquid nitrogen prior to data collection.

### Structure determination and refinement

The NR2E3 crystals formed in the P2 _1_2 _1_2 space group (Table 1). The datasets were collected with a MAR225 CCD detector at the ID line of sector 21 of the Advanced Photon Source at Argonne National Laboratory (Argonne, IL). The observed reflections were reduced, merged, and scaled with DENZO and SCALEPACK in the HKL2000 package [66].

The CCP4 program PHASER was used for molecular replacement [67], with the TR4 LBD (PBD code: 3P0U) [39] as a search model. The initial model was manually built and refined with CNS and the CCP4 program REFMAC [68]. All figures were prepared using PyMOL [69]. The pocket volume and surface area were calculated using VOIDOO [70].

During structure determination, molecular replacement solutions obtained from using MBP as an ensemble to search for 2 copies of MBP failed. We were only able to produce an interpretable electron density map for one MBP. Islands of electron density could be seen in the supposed area of the other MBP, but the density was not sufficient for us to solve the structure of a second MBP. However, the lack of clear density for the second MBP was not due protein truncation/proteolysis during crystallization as washed crystals loaded on a denaturing SDS-PAGE clearly showed the intact fusion protein (data not shown).

### Mammalian two-hybrid assay and luciferase reporter assays

The expression plasmids were constructed by inserting NR2E3-LBD (residues 192-410) into the pBind-GAL4 vector and pACT-VP16 vectors (Promega). Transfections were performed in AD293 cells, which were maintained in Dulbecco’s modified Eagle’s medium (DMEM) supplemented with 10% FBS. Cells were plated in 24-well tissue culture plates. After overnight incubation, cells were transfected with 100 ng of pBind-GAL4-NR2E3 LBD, 100 ng of pGLuc-5, and 0.1 ng of pHRL-TK-RLuc in Opti-MEM using Extreme9 according to the manufacturer’s protocol (Promega). One day after transfection, medium was changed to fresh medium. Two days after transfection, lysates were collected and luciferase activity was measured using the Dual Luciferase Reporter Assay System (Promega). Firefly luciferase values were normalized to Renilla luciferase as an internal transfection control. Luciferase activities were measured using an EnVision Multilabel Reader (PerkinElmer). All assays were performed in triplicate and presented with standard deviations.

### Extraction of protein samples and Western blotting

Protein extracts were prepared using the CelLytic MT Cell Lysis Reagent (Sigma) and protein concentrations were determined by Bradford Assay. Proteins from lysates were separated by 4-20% gradient SDS-PAGE, transferred to nitrocellulose membrane, and detected by immunoblotting using anti-Gal4 DBD antibodies (Santa Cruz; sc-510).

### Site Directed Mutagenesis

Point mutations were introduced in pGAL4-NR2E3-LBD and pACT-NR2E3-LBD constructs using the QuikChange method (Agilent). All mutant constructs were confirmed by DNA sequencing.

### Statistical Analysis

Statistical analysis was performed using Excel. Comparisons were performed using Student’s independent-sample t-test. The statistical significance level was set to be p < 0.05 or p < 0.01.
